# The serum proteomic profile in patients with migraine

**DOI:** 10.3389/fnmol.2025.1460403

**Published:** 2025-03-24

**Authors:** Yating Han, Yuan Wang, Xiajuan Zou, Huailian Guo

**Affiliations:** ^1^Department of Neurology, Peking University People’s Hospital, Beijing, China; ^2^Synthetic and Functional Biomolecules Center, Key Laboratory of Bioorganic Chemistry and Molecular Engineering of Ministry of Education, Beijing National Laboratory for Molecular Sciences, College of Chemistry and Molecular Engineering, Peking University, Beijing, China; ^3^Medical and Healthy Analysis Center, Peking University, Beijing, China; ^4^Key Laboratory for Neuroscience, Ministry of Education, National Health and Family Planning Commission, Peking University, Beijing, China

**Keywords:** ACTG1, ENO2, glycolysis, HIF-1 signaling pathway, migraine, proteomics

## Abstract

**Background:**

Migraine is a paroxysmal headache disorder, which seriously affects the patients’ quality of life. However, the pathogenesis of migraine is not clear yet. Proteomics is an emerging technology for studying small molecules and protein components in biological systems. This study aimed to analyze the serum proteome of migraine patients and healthy controls and identify differentially expressed proteins, which could provide a reference for the study of biomarkers and pathophysiological mechanisms of migraine.

**Methods:**

Fasting venous blood was collected, and serum was separated. Liquid chromatography–mass spectrometry was used to detect the proteome of the two groups, and MaxQuant was used to analyze the protein profile and identify the differentially expressed proteins.

**Results:**

Twenty-seven migraine patients and 20 healthy people matching the age and sex ratio of the migraine group were collected. A total of 27 differentially expressed proteins were identified between migraine and control groups, which were mainly related to immune response, inflammation, glycolysis, lipid metabolism, neurotrophy and development, and so on. Subgroup analysis also identified several differentially expressed proteins between the migraine with aura and the migraine without aura groups and between the ictal and interictal migraine groups. Moreover, the signal pathways that may be related to migraine include the glycolysis/gluconeogenesis pathway and the hypoxia-inducible factor-1 signal pathway. Differentially expressed proteins are mainly distributed in the extracellular area. Related biological processes include complement activation, immunoglobulin receptor binding, and phagocytosis.

**Discussion:**

The research screened out several differentially expressed proteins of migraine patients, which may be potential biomarkers, but it still needs verification in further studies with larger sample sizes. Various proteins related to inflammation, immune response, and energy metabolism are differentially expressed between the migraine group and the control group, suggesting that the pathogenesis of migraine may be related to inflammation, immunity, and energy metabolism disorders. In the future, we can further explore the therapeutic targets of migraine in terms of these biological processes.

## Introduction

Migraine is a paroxysmal headache disorder characterized by recurrent, mostly unilateral, pulsating, moderate-to-severe headaches, often accompanied by nausea, vomiting, photophobia, and phonophobia, which may also have vision, sensory, or other kinds of aura. Patients would suffer from headaches for 4 to 72 h without treatment or with ineffective treatment (Headache Classification Committee of the International Headache Society (IHS), 2018). Migraine is a common disease. The global prevalence of migraine is 14.7%, and the Chinese annual prevalence is 9.3% (Yu et al., 2011). The prevalence in women is two to three times more than men. The highest prevalence occurs between the ages of 25 and 55 years. According to the Global Burden of Disease Study by the World Health Organization in 2021, migraine was the third most disabling disease of neurological disorders in the world, which seriously affected the patients’ quality of life (GBD 2021 Nervous System Disorders Collaborators, 2024).

However, the pathophysiology of migraine is still incompletely understood. There are generally accepted mechanisms that may play a role in the migraine attack, including cortical spreading depression, alteration in thalamocortical circuits, altered brain connectivity, and sensitization of the trigemino-cervical complex (Charles, 2018). The diagnosis of migraine depends on clinical history at present. Although well-accepted diagnostic criteria exist for migraine, it is still a complex disorder that remains both underdiagnosed and misdiagnosed. The lack of appropriate biomarkers is an obstacle to developing more effective diagnostic criteria and treatments (Durham and Papapetropoulos, 2013).

Proteomics studies the expression and function of proteins in a biological system, together with the pathophysiological processes in which they are involved, helping to define more precisely the pathogenesis of diseases. Currently, proteomics is one of the most significant methodologies for the identification of the overall protein content of cells, tissues, or biofluids (Lionetto et al., 2013; Hasin et al., 2017; Hosp and Mann, 2017). Compared to genomics or metabolomics, proteomics provides a new perspective to explore the mechanism and biomarker of migraine. Moreover, proteins are the direct reflection of biological function, and the occurrence and development of many diseases are caused by the abnormal expression or dysfunction of proteins, rather than the genes. Therefore, the proteomic approach could be appropriate to explore promising biomarkers of multifactorial diseases such as migraine. However, at present, few proteomics studies have been developed in an attempt to identify biomarkers and pathophysiological mechanisms associated with migraine. Klychnikov et al. (2010) focused on the cortical synapse proteomics of a transgenic migraine mouse model with mutated CaV2.1 calcium and observed some upregulated proteins in the mutant mice related to neurite outgrowth and actin dynamics, vesicle turnover, and glutamate transporters. Guyuron et al. (2014) compared the protein expression of the zygomaticotemporal branch of the trigeminal nerve in patients with and without migraine headaches and found five significant pathways involved in cytoskeletal organization, myelination of axons, and nervous system development. Bellei et al. (2020, 2021) examined the serum and urinary proteome of females suffering from menstrually related migraine and postmenopause migraine, in the search for potential biomarkers.

This study aimed to analyze the serum proteome of migraine patients in comparison with healthy controls and to search and identify the differentially expressed proteins as potential biomarkers of migraine, which could be informative on the accurate diagnosis and treatment of migraine.

## Materials and methods

### Study cohorts

**Figure 1:**
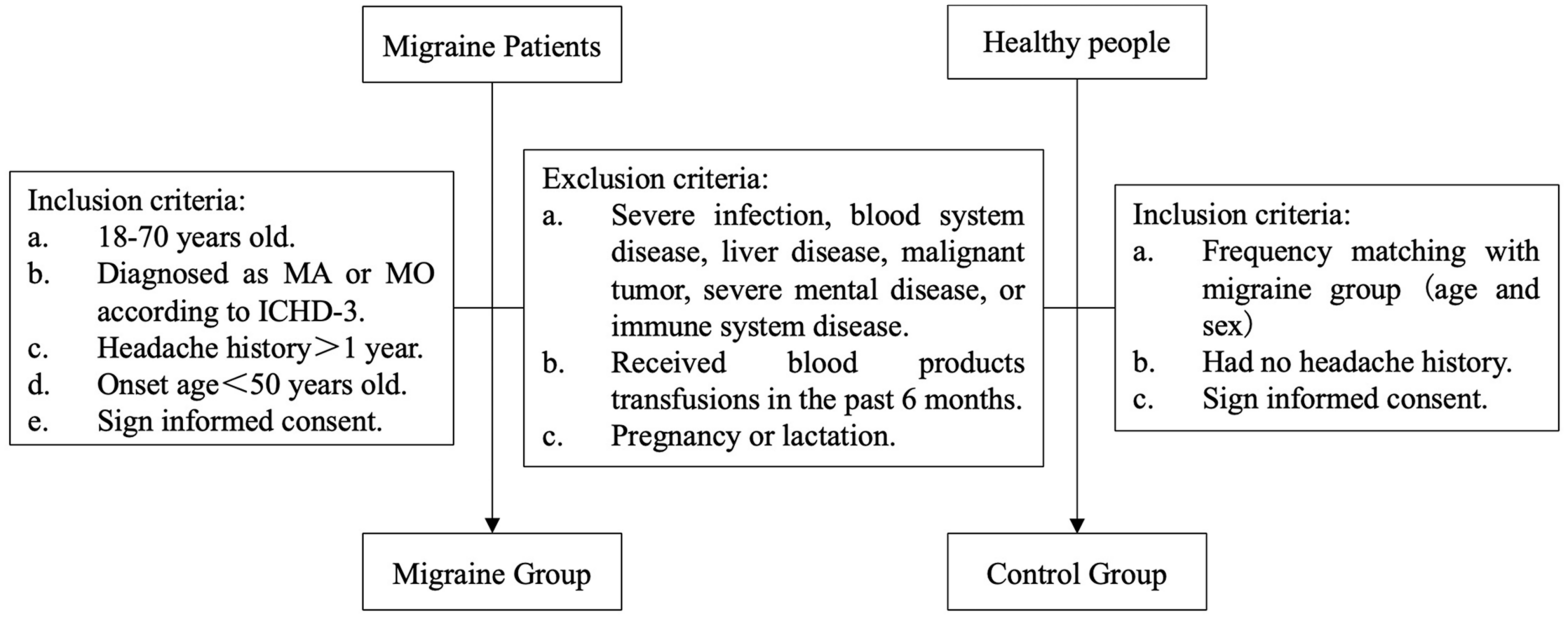
Screening criteria of the migraine and control groups.

In this study, participants who were diagnosed as migraine without aura or migraine with aura according to the diagnostic criteria of the International Classification of Headache Disorders, third edition (ICHD-3) and had a headache history of more than 1 year were included as the migraine group. Healthy adults who were age-matched and sex-matched with the migraine group and had no headache were included as the control group. Patients and healthy adults with the following conditions were excluded: (1) with severe infection, blood system disease, liver disease, malignant tumor, severe mental disease, or immune system disease, (2) received blood products transfusions in the past 6 months, and (3) with pregnancy and lactation (Figure 1). The baseline demographic data and clinical history were collected through questionnaires. All participants gave their written informed consent for inclusion before they participated in the study. The study was approved by the Medical Ethics Committee of the Second Clinical Hospital of Peking University.

### Blood sample collection and serum preparation

Fasting venous blood (4 mL) was collected in the morning from migraine patients and non-headache controls. Blood samples were collected into 4 mL BD Biosciences gold top and placed on ice. Serum was subsequently subjected to centrifugation at 2,000–3,000×*g* for 10 min at +4°C. To preserve proteins, serum was added with a protease inhibitor cocktail (Roche.) in a ratio of 1:500 prior to being divided into aliquots and stored at −80°C until proteomic analysis.

### Sample preparation for proteomics

The serum proteins were digested according to the manufacturer’s protocol for filter-aided sample preparation (FASP) with minor modifications. The serum (10 μL) was diluted in 8 M urea (90 μL). The mixture was heated at 95°C for 10 min. When the mixture was left to cool to room temperature, tris-(2-carboxyethyl) phosphine (TCEP) was added to the serum solution to a final concentration of 10 mM and heated at 67°C for 10 min. Then, 50 mM iodoacetamide (IAA) was added to the alkylated solution. After the sample was incubated at room temperature for 30 min in the dark, all of the samples were transferred to the Vivacon 500 filtration tubes (Cat No. VNO1HO2, Sartorius Stedim Biotech, UK, MWCO 10 kDa), mixed with 150 μL of 8 M urea in 0.1 M Tris/HCl (pH 8.5), and centrifuged at 14,000 g for 15 min at 20°C. This step was performed two times. Then, the samples were washed three times with 100 μL of 50 mM NH_4_HCO_3_. Finally, 6 μg of trypsin (Promega, Madison, WI) in 100 μL of 50 mM NH_4_HCO_3_ was added to each filter. The protein-to-enzyme ratio was 100:1. The samples were incubated overnight at 37°C. Released peptides were collected by centrifugation and vacuum-dried.

### Liquid chromatography–mass spectrometry (LC–MS) analysis for proteomics

After redissolving with 0.1% FA, the peptide samples (1 μg) were analyzed by an LC–MS/MS system consisting of UltiMate 3,000 UHPLC (Thermo Scientific) and Q-Exactive HF (Thermo Fisher Scientific). The peptides were loaded on a homemade pre-column (2 cm × 100 μm i.d., 15–20 μm, 300 A, YWG-C18) before being separated on a homemade analytical column (12 cm × 75 μm i.d., 15–20 μm, 300 A, YWG-C18) in the 65°C thermostat at a flow rate of 400 nL/min. For a gradient separation, H_2_O/FA (99.9:0.1) was used as mobile phase A, whereas ACN/H_2_O/FA (80:19.9:0.1) was used as mobile phase B. The LC gradient began with 5% phase B for 5 min and ramped to 10% for 3 min, from 10 to 40% for 92 min, and then from 40 to 100% for 10 min; it was held at 100% for 4 min. Finally, the concentration was restored to 5% phase B and held for 6 min before the next run.

MS/MS spectra were obtained in a data-dependent high-energy collision dissociation (HCD) model. The parameters for serum peptide analysis were as follows: (1) full MS: scan range = 350–1,500 m/z; resolution = 60,000; AGC target = 3,000,000; maximum injection time = 20 ms; included charge state = 2–6; dynamic exclusion duration = 30 s; (2) dd-MS2: isolation window = 1.6 m/z; resolution = 15,000; AGC target = 100,000; maximum injection time = 45 ms; loop count = 20; stepped collision energy = 27.

### Database search and statistical analysis

Raw data were analyzed using the MaxQuant software (version 1.5.6.0). MS data were searched against the UniProt Human database, with the following settings: Carbamidomethylation of cysteine was defined as fixed modification and methionine oxidation (+15.99 Da) as a variable modification of the peptides. Trypsin was set as the protease with two missed cleavage sites allowed. Label-free quantification (LFQ) can calibrate the raw intensity values between samples to eliminate experimental batch effects or technical errors between samples and finish data normalization. LFQ was performed in MaxQuant following that the minimum ratio count for LFQ was set to 2 and the match-between-runs option was enabled. Other parameters were set as default. The continuous variables normally distributed were expressed as mean ± standard deviation (SD) and the dichotomous variables as percentages. The false discovery rate was set as less than 0.01. The comparison between means was made using a *t*-test. Differences were considered significant with a *p*-value <0.05. Regarding the fold changes of spot abundance, values >1.2 were considered as significant.

All statistical analyses were performed using SPSS 27.0. Values are presented as the mean ± standard deviation for data that were normally distributed or median and interquartile range for data that were not normally distributed for continuous variables and number (%) for categorical variables. For two-group comparison, *p*-values were derived from the one-way Student *t*-test to determine the differences between groups with the normally distributed data and Mann–Whitney non-parametric test with other data. For all comparisons, a *p-*value of <0.05 was considered statistically significant.

## Results

### Characteristics of participants

A total of 27 migraine patients and 20 healthy controls were enrolled in this study. There was no statistical difference in gender ratio and average age between the two groups. The BMI of the migraine group was significantly lower than that of the control group, and the proportion of allergic diseases and the SAS scale score were significantly higher than those of the control group (Table 1).

**Table 1:** Demographic characteristics of participants.

Characteristics	Migraine group (*n* = 27)	Control group (*n* = 20)	*P*-value
	*N* (%)	*N* (%)	
Sex (male/female)	9/18	9/11	0.416
Mean age (years)	40.5 ± 10.9	39.5 ± 11.0	0.756
BMI (kg/m^2^)	22.03 ± 2.06	24.58 ± 3.05	0.002*
Ictal phase	7 (25.93)	/	
Migraine with aura	5 (18.52)	/	
Family history of headache	13 (48.15)	/	
VAS(>6)	19(70.37)	/	
Acute migraine treatment			
Triptans	2 (7.41)	/	
NSAIDs	14 (51.85)	/	
Analgesic combination	9 (33.33)	/	
Comorbidities			
Hypertension	1 (3.70)	1 (5)	0.828
Hyperlipidemia	4 (14.81)	2 (10)	0.625
Allergic disease	12 (44.44)	3 (15)	0.032*
Scale scores			
SAS	39.24 ± 7.07	34 ± 5.46	0.011*
SDS	39.12 ± 9.94	35.25 ± 7.42	0.164

*p < 0.05. BMI, body mass index; VAS, visual analog scale; NSAIDs, non-steroidal anti-inflammatory drugs; SAS, Self-Rating Anxiety Scale; SDS, Self-Rating Depression Scale.

### Differentially expressed proteins between migraine patients and healthy controls

**Figure 2:**
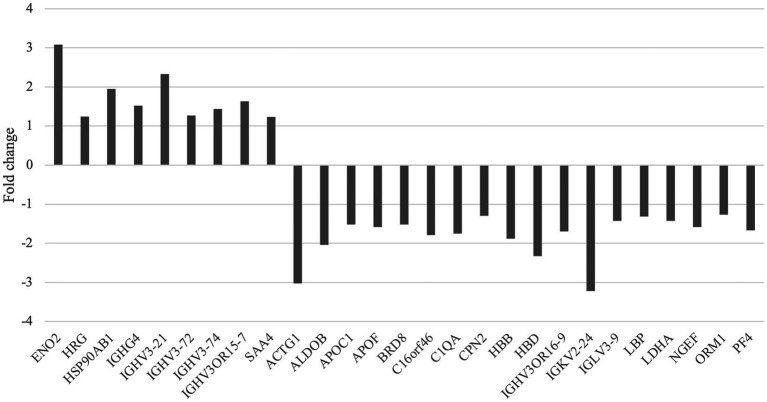
Upregulated and downregulated proteins of the migraine group compared with the control group.

**Figure 3:**
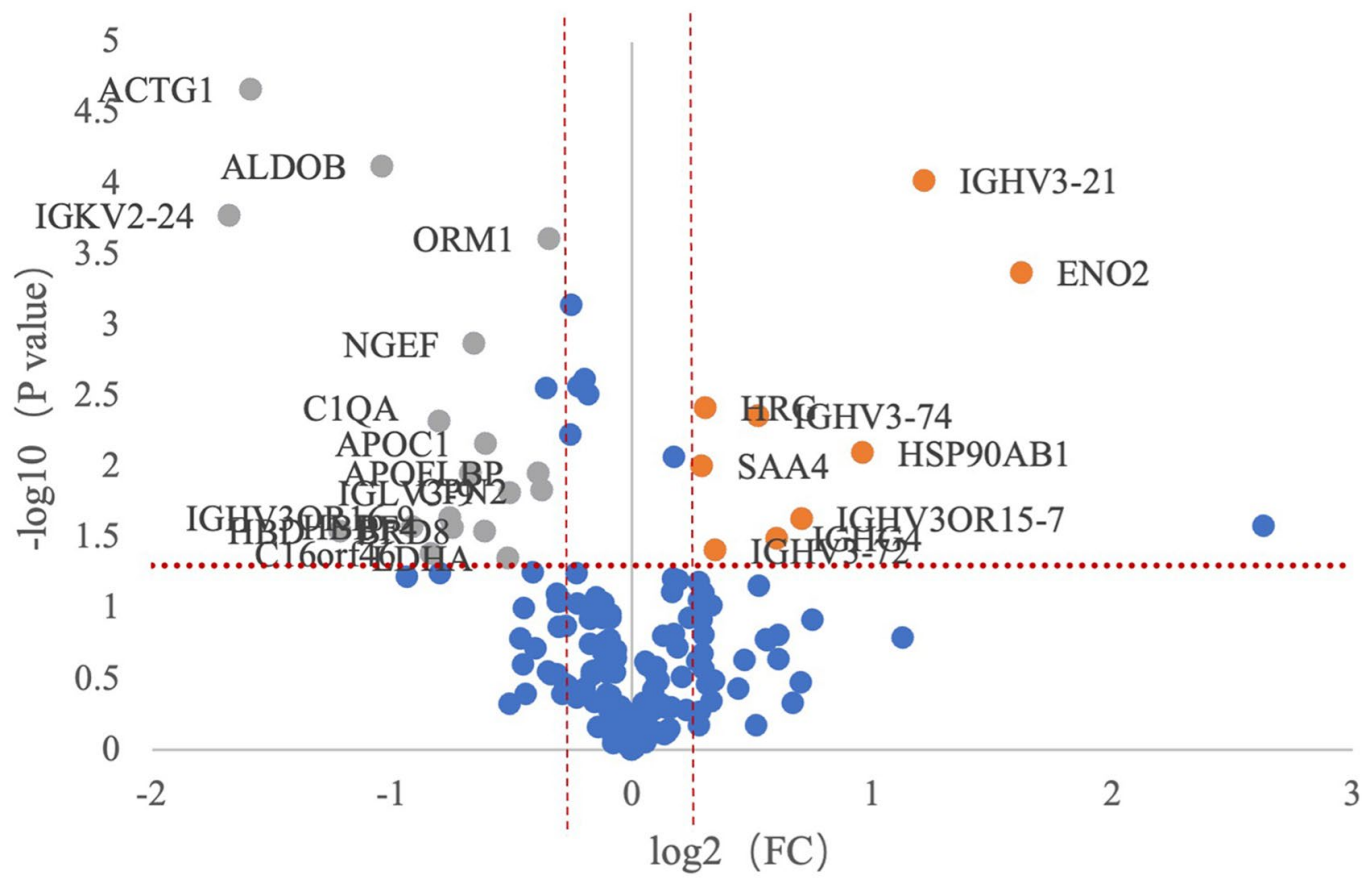
Volcano plot demonstrating differentially expressed proteins of the migraine group compared with the control group.

There were 27 differentially expressed proteins identified between the migraine and control groups (Table 2). Compared with healthy controls, nine proteins were upregulated, which were involved in neurotrophy, neuroprotection, molecular chaperone, immunity, and inflammation, and 18 were downregulated, which were related to cell motility, glycolysis, lipid metabolism, immunity, neurogenesis, and so on (Figures 2, 3).

**Table 2:** Differentially expressed proteins between migraine patients and healthy controls.

Protein full name	Gene name	Fold change	*P*-value (*t*-test)	Change	Function
Gamma-enolase	ENO2	3.08	0.000	Up	Neurotrophy, neuroprotection
Histidine-rich glycoprotein	HRG	1.24	0.004	Up	Multifunction
Heat shock protein HSP 90-beta	HSP90AB1	1.95	0.008	Up	Molecular chaperone
Immunoglobulin heavy constant gamma 4	IGHG4	1.52	0.032	Up	Immunity
Immunoglobulin heavy variable 3-21	IGHV3-21	2.33	0.000	Up	Immunity
Immunoglobulin heavy variable 3-72	IGHV3-72	1.27	0.039	Up	Immunity
Immunoglobulin heavy variable 3-74	IGHV3-74	1.44	0.004	Up	Immunity
Immunoglobulin heavy variable 3/OR15-7	IGHV3OR15-7	1.63	0.023	Up	Immunity
Serum amyloid A-4 protein	SAA4	1.23	0.010	Up	Inflammation
Actin, cytoplasmic 2	ACTG1	0.33	0.000	Down	Cell motility
Fructose-bisphosphate aldolase B	ALDOB	0.49	0.000	Down	Glycolysis
Apolipoprotein C-I	APOC1	0.66	0.007	Down	Lipid metabolism
Apolipoprotein F	APOF	0.63	0.011	Down	Lipid metabolism
Bromodomain-containing protein 8	BRD8	0.66	0.029	Down	Transcription regulation
Uncharacterized protein C16orf46	C16orf46	0.56	0.041	Down	Unknown
Complement C1q subcomponent subunit A	C1QA	0.57	0.005	Down	Immunity
Carboxypeptidase N subunit 2	CPN2	0.77	0.015	Down	Immunity
Hemoglobin subunit beta	HBB	0.53	0.027	Down	Oxygen transport
Hemoglobin subunit delta	HBD	0.43	0.028	Down	Oxygen transport
Immunoglobulin heavy variable 3/OR16-9	IGHV3OR16-9	0.59	0.023	Down	Immunity
Immunoglobulin kappa variable 2-24	IGKV2-24	0.31	0.000	Down	Immunity
Immunoglobulin lambda variable 3-9	IGLV3-9	0.70	0.015	Down	Immunity
Lipopolysaccharide-binding protein	LBP	0.76	0.011	Down	Immunity
L-lactate dehydrogenase A chain	LDHA	0.70	0.044	Down	Glycolysis
Ephexin-1	NGEF	0.63	0.001	Down	Neurogenesis
Alpha-1-acid glycoprotein 1	ORM1	0.79	0.000	Down	Inflammation
Platelet factor 4	PF4	0.60	0.027	Down	Platelet aggregation

**Figure 4:**
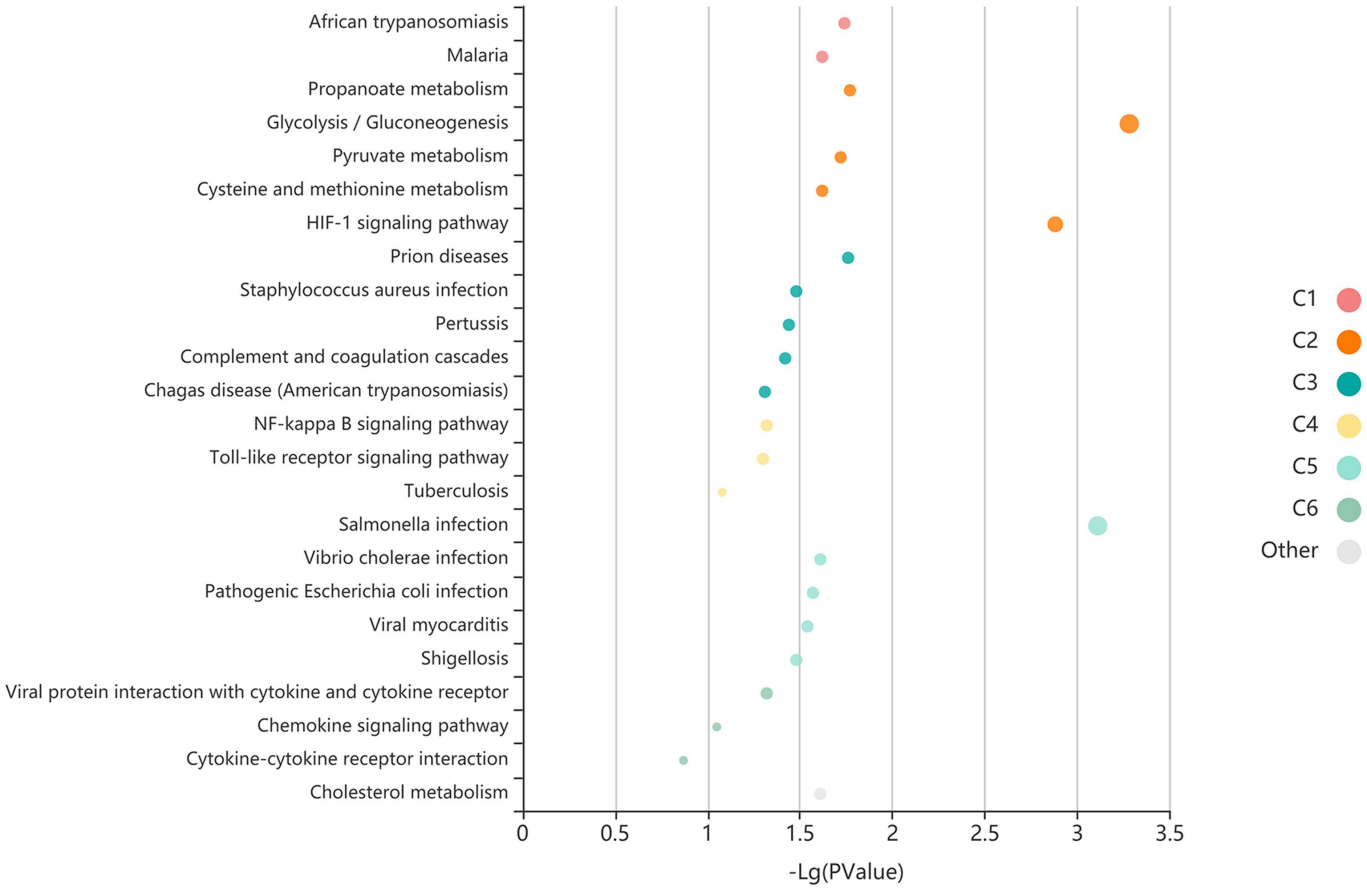
Bubble plot of signal pathways related to migraine.

We performed a search of these 27 proteins in the Gene Ontology (GO) and Kyoto Encyclopedia of Genes and Genomes (KEGG) database (Table 3). The results of GO and KEGG analysis indicated that the signal pathways that might be related to migraine included the glycolysis/gluconeogenesis pathway and the hypoxia-inducible factor-1 (HIF-1) signal pathway (Figure 4). Differentially expressed proteins were mainly distributed in the extracellular area. Related biological processes included complement activation, immunoglobulin receptor binding, and phagocytosis.

**Table 3:** GO and KEGG analysis of differentially expressed proteins between the migraine and control groups.

Database	Term	*P*-value	Corrected *p*-value
KEGG PATHWAY	Glycolysis/gluconeogenesis	1.61E-05	3.14E-04
	HIF-1 signaling pathway	6.28E-05	8.99E-04
Gene Ontology	Complement activation, classical pathway	1.54E-11	4.05E-09
	Immunoglobulin complex, circulating	4.26E-11	4.05E-09
	Immunoglobulin receptor binding	5.87E-11	4.05E-09
	Positive regulation of B-cell activation	6.24E-11	4.05E-09
	Phagocytosis, recognition	6.64E-11	4.05E-09
	Phagocytosis, engulfment	1.73E-10	8.78E-09
	B-cell receptor signaling pathway	2.98E-10	1.30E-08
	Antigen binding	6.34E-10	2.33E-08
	Blood microparticle	6.89E-10	2.33E-08
	Defense response to bacterium	6.75E-09	2.06E-07
	Extracellular space	4.93E-08	1.37E-06
	Innate immune response	8.51E-08	2.16E-06
	External side of plasma membrane	2.58E-07	6.05E-06
	Extracellular exosome	6.67E-07	1.36E-05
	Extracellular region	2.45E-06	4.39E-05
	Cytosol	5.43E-03	2.12E-02
	Protein binding	3.62E-02	5.45E-02

GO, Gene Ontology; KEGG, Kyoto Encyclopedia of Genes and Genomes; HIF-1, hypoxia-inducible factor 1.

### The proteome profiles of migraine subgroups

Compared with the migraine without aura (MO) group, migraine with aura (MA) group had eight differentially expressed proteins, of which seven were upregulated and one was downregulated, which were involved with oxygen transport, immunity, blood coagulation, cell adhesion, and lipid metabolism (Table 4).

**Table 4:** Differentially expressed proteins between the MA and MO groups.

Protein full name	Gene name	Fold change	*P*-value (*t*-test)	Change	Function
Hemoglobin subunit delta	HBD	2.47	0.000	Up	Oxygen transport
Complement component C8 alpha chain	C8A	1.22	0.012	Up	Immunity
Thyroxine-binding globulin	SERPINA7	1.25	0.016	Up	Thyroid hormone transport
Hyaluronan-binding protein 2	HABP2	1.47	0.026	Up	Coagulation
					Cell adhesion
Apolipoprotein L1	APOL1	1.29	0.028	Up	Lipid metabolism
Lipopolysaccharide-binding protein	LBP	1.41	0.040	Up	Immunity
Endoribonuclease	ERN1	1.55	0.041	Up	Transcription regulation
					Cellular response to stimulus
Thrombospondin-1	THBS1	0.66	0.034	Down	Cell migration
					Inflammatory response

Compared with the interictal migraine group, there were four differentially expressed proteins in the ictal migraine group, of which two were upregulated and two were downregulated, which were related to inflammation and immunity (Table 5).

**Table 5:** Differentially expressed proteins between the ictal and interictal groups.

Protein full name	Gene name	Fold change	*P*-value (*t*-test)	Change	Function
Alpha-1-acid glycoprotein 2	ORM2	1.26	0.004	Up	Inflammation
Mannose-binding protein C	MBL2	1.60	0.039	Up	Immunity
Immunoglobulin heavy variable 1-18	IGHV1-18	0.66	0.031	Down	Immunity
Immunoglobulin heavy variable 4-31	IGHV4-31	0.67	0.031	Down	Immunity

Among migraine patients, compared with patients who had no family history of headaches, three proteins were upregulated and four proteins were downregulated in the patients who had a family history of headaches, which were related to embryonic organ development, coagulation, immunity, cell adhesion, and cell movement (Table 6).

**Table 6:** Differentially expressed proteins between migraine patients with and without headache family history.

Protein full name	Gene name	Fold change	*P*-value (*t*-test)	Change	Function
Retinol-binding protein 4	RBP4	1.31	0.029	Up	Embryonic organ development
Immunoglobulin lambda variable 3–25	IGLV3-25	1.46	0.033	Up	Immunity
Protein Z-dependent protease inhibitor	SERPINA10	1.59	0.017	Up	Coagulation
Complement component C7	C7	0.78	0.013	Down	Immunity
Platelet basic protein	PPBP	0.81	0.016	Down	Immunity
Fibronectin	FN1	0.78	0.023	Down	Cell adhesion
					Cell motility
Immunoglobulin heavy variable 1–69-2	IGHV1-69-2	0.61	0.042	Down	Immunity

## Discussion

Migraine is a common primary headache in clinical practice, whose clinical manifestations are episodic moderate-to-severe pulsatile headache. According to the Global Burden of Disease Study, migraine is the most disabling neurological disease among the ages 5–19 years and second after stroke among adults aged 20–59 years (Peres et al., 2024). Frequent migraine attacks and chronic migraine have a serious impact on the study, work, and daily life of migraine patients (Deuschl et al., 2020). Currently, it is believed that migraine is a chronic neurovascular disease that involves multiple pathogenesis mechanisms such as genetics, immunity, endocrine, and metabolism. However, looking for suitable biomarkers to assist in the diagnosis and treatment of migraine still remains an urgent issue to be addressed.

In this study, a total of 27 differential proteins between migraine patients and healthy controls were identified by liquid chromatography–mass spectrometry. Among these 27 proteins, eight belong to the component of immunoglobulins, and C1QA, LBP, HRG, ORM1, and SAA4 are also related to inflammation and immunity. The differential proteins between the ictal and the interictal groups are also related to inflammation and immunity, suggesting that neuroinflammation and immune disorders may participate in the pathophysiological mechanism of migraine. Previous migraine proteomics studies had similar findings. Similar to our study, Woldeamanuel et al. (2024) performed untargeted proteomics and found proteins for classifying migraine patients with migraine from healthy controls, including immunoglobulin heavy variable 3–74 (IGHV 3–74). Bellei et al. (2020, 2021) observed that IGKC and CO4A levels were higher in the serum of women with menstrual-related migraine and postmenopausal migraine, which was also found in the urine proteomics study of the same participants. Togha et al. (2022) analyzed proteomics changes of migraine patients compared to controls or their pain-free period and found that the expression levels of several proteins such as haptoglobin, clusterin, and fibrinogen alpha chain showed considerable changes, and most of them were associated with inflammation, oxidative stress, and neuroprotection. The discrepancies in migraine proteomics studies may be related to sample heterogeneity, such as different tissue and disease courses, and different methods of sample preparation and proteomics techniques.

Some evidence supporting the role of immune disorders in the pathogenesis of migraine exists. Studies have found changes in cytokine levels and lymphocyte subsets in migraine patients (Perini et al., 2005; Aydn et al., 2015; Arumugam and Parthasarathy, 2016). In addition, migraine and autoimmune diseases have been found to have common genetic and environmental pathogenic factors. Polymorphisms in genes encoding human leukocyte antigen (HLA) and cytokines are considered risk factors for autoimmune diseases. Some studies have found that these genes are also involved in the pathogenesis of migraine (Rainero et al., 2005; Yilmaz et al., 2010).

*γ*-Enolase, also known as neuron-specific enolase (NSE), is an enzyme located in the axons and cytoplasm of neurons and plays a key role in glycolysis. Its elevated concentration in serum indicated neuronal damage caused by stroke or brain injury (Teepker et al., 2009). This study found that the serum NSE level in migraine patients increased, which suggested that migraine might be related to the injury of glial cells or neurons in the brain and damage to blood–brain barrier. Previous studies found inconsistent results. Yilmaz (2020) found that the serum NSE level in migraine patients was lower than that in controls, whereas Gönen et al. (2021) observed that the serum NSE level in both paroxysmal and chronic migraine patients was significantly higher than that in the control group. Yilmaz et al. (2011) found that the serum NSE level in MO patients during the ictal period was higher than that in the control group. Other studies have shown that there is no significant difference in serum NSE levels in children between the migraine and control groups (Azapağasi et al., 2012).

Compared with healthy controls, the levels of APOC1 and APOF in migraine group decreased, and the levels of APOL1 in MA patients increased compared with MO patients, indicating that lipid metabolism disorders may be related to the pathogenesis of migraine. Previous studies about lipidomics have found abnormal levels of lipids and apolipoprotein in migraine patients (Ren et al., 2018; Onderwater et al., 2019). Dyslipidemia is known as a vascular risk factor, and the incidence rate of cardiovascular and cerebrovascular diseases in migraine patients has also increased, but the specific relationship between migraine and dyslipidemia is still unclear.

We found two signal pathways based on 27 differentially expressed proteins, including the glycolysis/gluconeogenesis pathway and HIF-1 signal pathway, indicating that the pathogenesis of migraine is related to energy metabolism and oxidative stress. Liu et al. (2022) observed that joint pathway analysis of proteomics and metabolomics revealed significant enrichment of the pentose phosphate and glycolysis/gluconeogenesis pathways, which provided evidence for the influence of energy metabolism on migraine. Previous studies have suggested that migraine is a stress response to brain energy deficiency and oxidative stress. Some researchers used magnetic resonance spectroscopy (MRS) to detect ATP, lactic acid, magnesium, and other substances in the brains of migraine patients. It has been found that oxidative phosphorylation of mitochondria in migraine patients’ brains is impaired, manifested by increased ADP levels, decreased organic phosphate, and free magnesium levels (Gross et al., 2019). These findings suggest that the pathogenesis of migraine may be related to the imbalance of energy and glucose metabolism in the brain.

Compared with the control group, multiple proteins were significantly upregulated or downregulated in the migraine group, and subgroup analysis also showed that there was a differential expression of proteins between the ictal and interictal period of migraine between MO and MA as well as between with and without family history of headache. These proteins may be potential biomarkers of migraine, but further validation experiments are needed. The differential expression of various proteins related to inflammation, immune response, and energy metabolism suggests that the pathogenesis of migraine may be related to inflammation, immunity, and energy metabolism disorders. Specific molecular mechanisms can be researched in the future.

This study has some limitations. At first, the sample size was small. Further study needs to be performed in larger-sized populations. Second, the differentially expressed proteins identified through proteomics need to be further confirmed by enzyme-linked immunosorbent assay (ELISA) or Western blot. Because of the long storage time of original serum samples, new samples need to be collected for further validation. In addition, the signal pathways related to migraine need to be deeply studied. To further validate the involvement of the HIF-1 pathway and inflammation in migraine, we plan to establish a migraine model in mice by repeated injection of nitroglycerin (10 mg/kg, i.p.). Roxadustat, a HIF-1*α* stabilizer, will be orally administered before or after nitroglycerin injection. Mice will be randomly divided into three groups in the prevention and acute treatment experiments: the normal saline control group (NS), the NTG-induced migraine group (NTG), and the NTG plus roxadustat treatment group (NTG + Rox). Pressure application measurement and tail flick and light-aversive behavior tests will be performed to determine the pressure pain threshold, thermal nociceptive sensitivity, and migraine-related light sensitivity. At the end of experiments, mouse serum samples and brain tissues will be collected to analyze the expression of HIF-1α and proinflammatory, including factors interleukin (IL)-1β and IL-6 and tumor necrosis factor (TNF)-α. The expected results include that compared with the NS group, mice in the NTG group show a significant decrease in both the pressure pain threshold and thermal nociceptive threshold after NTG injection, and prevention or treatment with roxadustat can improve NTG-induced light sensitivity. HIF-1α will be reduced by NTG injection and be enhanced by roxadustat. In addition, roxadustat can improve the neuroinflammation induced by the repeated administration of NTG, as evidenced by the decreased protein and mRNA levels of proinflammatory factors. In a word, the hypothesis of further study is that HIF-1α stabilization can improve migraine-like behaviors and inhibits inflammation in nitroglycerin-injected mice, which may be mediated by the HIF-1α/NF-κB/inflammation pathway, suggesting the potential of HIF-1α activators as therapeutics for migraine.

In conclusion, this study could provide some new insights into the treatment of migraine. We can further explore therapeutic targets of migraine in the fields of inflammation, immunity, and energy metabolism in the future.

## Data Availability

The datasets presented in this study can be found in online repositories. The names of the repository/repositories and accession number(s) can be found in the article/Supplementary material.
